# Palladium Catalyzed Allylic C-H Alkylation: A Mechanistic Perspective

**DOI:** 10.3390/molecules16010951

**Published:** 2011-01-21

**Authors:** Casper Junker Engelin, Peter Fristrup

**Affiliations:** Department of Chemistry, Technical University of Denmark, Kemitorvet building 201, DK-2800, Kgs. Lyngby, Denmark

**Keywords:** palladium, C-H activation, allylic alkylation, catalysis

## Abstract

The atom-efficiency of one of the most widely used catalytic reactions for forging C-C bonds, the Tsuji-Trost reaction, is limited by the need of preoxidized reagents. This limitation can be overcome by utilization of the recently discovered palladium-catalyzed C-H activation, the allylic C-H alkylation reaction which is the topic of the current review. Particular emphasis is put on current mechanistic proposals for the three reaction types comprising the overall transformation: C-H activation, nucleophillic addition, and re-oxidation of the active catalyst. Recent advances in C-H bond activation are highlighted with emphasis on those leading to C-C bond formation, but where it was deemed necessary for the general understanding of the process closely related C-H oxidations and aminations are also included. It is found that C-H cleavage is most likely achieved by ligand participation which could involve an acetate ion coordinated to Pd. Several of the reported systems rely on benzoquinone for re-oxidation of the active catalyst. The scope for nucleophilic addition in allylic C-H alkylation is currently limited, due to demands on pK_a_ of the nucleophile. This limitation could be due to the pH dependence of the benzoquinone/hydroquinone redox couple. Alternative methods for re-oxidation that does not rely on benzoquinone could be able to alleviate this limitation.

## 1. Background

**Scheme 1:**
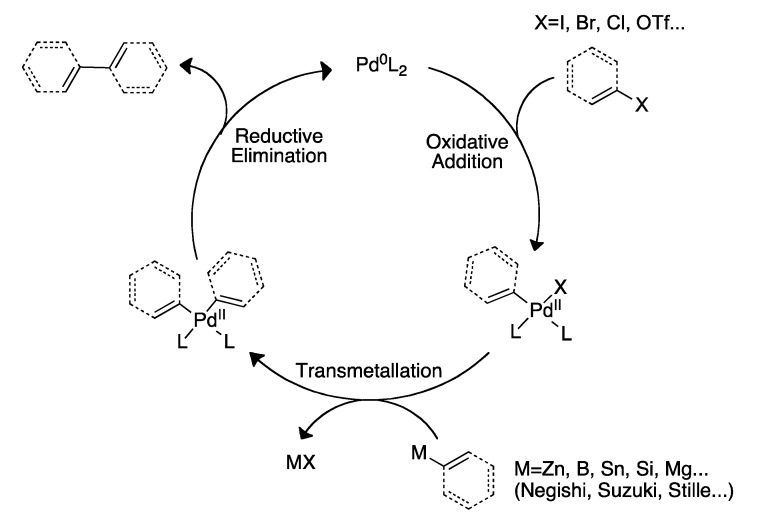
Simplified catalytic cycle for classical Pd-catalyzed cross-coupling reactions.

The forging of C-C bonds through palladium catalysis is widely used in chemistry and has recently been highlighted further by the 2010 Nobel Prize to Richard F. Heck, Ei-ichi Negishi and Akira Suzuki for their ground-breaking discoveries of Pd-catalysis leading to C-C bond formation [1]. The fundamental investigations which laid the foundation for these novel transformations were carried out in the 1960s and 1970s, however the reactions have remained among the most widely used ever since which can be attributed to a constantly evolving scope. The traditional cross-coupling reactions based on Pd-catalysis all require a preoxidized coupling partner, and the first step in the catalytic cycle thus involves an oxidative addition to Pd^0^ leading to the formation of a Pd^II^-intermediate (Scheme 1) [2]. 

**Scheme 2:**
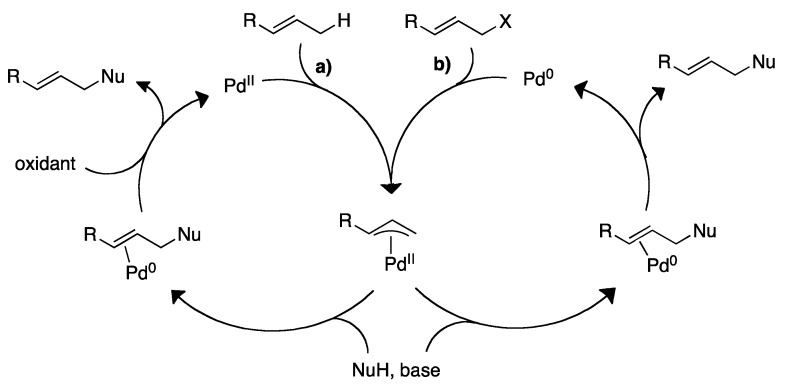
**a)** Allylic C-H alkylation and **b)** the Tsuji-Trost allylic alkylation. X = Br, Cl, OCOR, OCO_2_R, SO_2_R, P(=O)(OR)_2_, *etc*. NuH = *β*-dicarbonyls, enamines, enolates, etc. [11].

This is also the case with one of the most used palladium catalyzed reactions for organic synthesis of C-C bonds with introduction of chirality, namely the Tsuji-Trost reaction [3,4]. This reaction utilizes a broad range of allylic substrates with different leaving groups, such as acetates, halides, carbonates, epoxides, and phosphonates. The reaction also tolerates a wide range of nucleophiles, such as *β*-dicarbonyls, enamines, and enolates (Scheme 2). The success of this transformation can be ascribed to the fact that it generally displays a high level of chemo-, regio-, and stereoselectivity [5]. This selectivity can to a great extent be *predicted* beforehand using a series of working models and mnemonic devices developed by Trost and coworkers [6,7,8]. This prediction has been even more firmly linked to the structure of the catalyst with a new general model for selectivity in the asymmetric allylic alkylation of cycloalkenyl esters employing the Trost ‘Standard Ligand’ (TSL), as reported recently by Lloyd-Jones and coworkers [9]. This model involves two chemically distinct moieties for nucleofuge binding (NH) and nucleophile binding (CO). However, despite the overwhelming success of the classical Tsuji-Trost reaction, the atom efficiency [10] of the overall transformation is hampered by the necessity of introducing a leaving group. 

An alternative activation of the allylic coupling partner in the form of C-H activation can be possible under certain reaction conditions (Scheme 2). This modification greatly improves the atom-efficiency of the overall C-C bond formation as the preceding introduction of the leaving group is eliminated. The further development of a general method for allylic C-H activation followed by alkylation would greatly enhance the scope of the Tsuji-Trost alkylation [11]. 

**Scheme 3:**
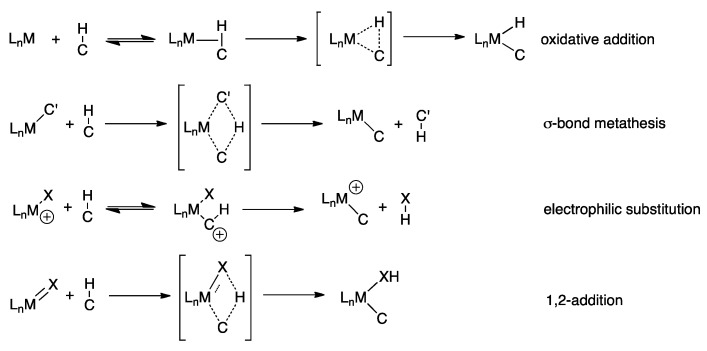
The different mechanisms for C-H activation [12].

Several mechanisms can account for the activation of a C-H bond and Eisenstein and coworkers has proposed four different mechanisms (Scheme 3) [12]. The first and most common mechanism is an oxidative addition, which is initiated by coordination of the C-H bond to a vacant site on the metal and results in the formation of a M-C and a M-H bond. Shilov and Shul’pin define this as “true” metal complex activation of the C-H bond, since the closest contact between a metal ion and the C-H bond is obtained in this scenario [13]. This mechanism is typical for electron-rich late transition metals, since the higher oxidation state and the change in geometry during the mechanism are not energetically unfavorable. For early transition metals the σ-bond metathesis mechanism is favored and characterized by a concerted forming and breaking of bonds in the transition state of the reaction. It is also a possibility that the metal acts as a Lewis acid and the hydrogen atom of the reagent is substituted by the metal. This mechanism is categorized as electrophilic substitution. Lastly the C-H bond can react with an unsaturated M-X bond in a 1,2 addition and the C-M bond is created without the breaking of the M-X σ-bond, as in the σ-bond metathesis. 

White [14] and Shi [15] recently published results concerning catalytic, intermolecular allylic C-H alkylation, which constitutes a breakthrough in this research area. The mechanism behind the long known Tsuji-Trost alkylation has been studied in detail [16], but this new allylic C-H alkylation is to date not fully mechanistically understood. In this review we will summarize recent results on mechanistic investigations of Pd-catalyzed C-H activation with the aim of achieving an overall understanding of the allylic C-H alkylation reaction. Where appropriate we will also include results obtained in related Pd-catalyzed transformations, such as C-H oxidation and amination. 

## 2. The Palladium Catalyzed Allylic C-H Alkylation

**Scheme 4:**
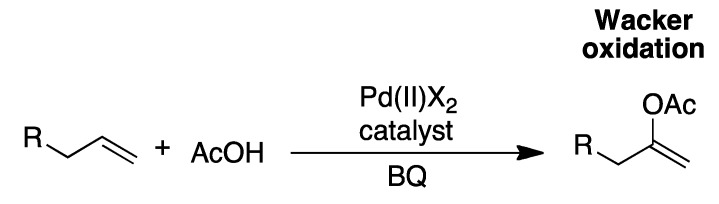
The formation of the product from Wacker oxidation [18].

Trost and Fullerton showed in 1973 that non-functionalized alkenes could undergo allylic alkylation, but this required a stoichiometric amount of Pd^II^ [17]. While this discovery had important mechanistic implications it was of little practical use as non-catalytic use of precious metals is not cost-effective. The catalytic version requires reaction conditions that support the Pd^II^-mediated electrophilic C-H cleavage, the nucleophilic attack and the reoxidation of Pd^0^ to Pd^II^. One of the difficulties with these steps is the formation of Wacker-type products (Scheme 4) [18,19]. 

**Scheme 5:**
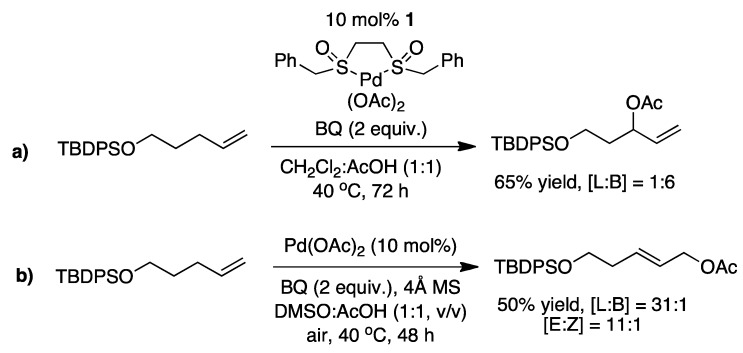
**a)** The palladium catalyzed allylic oxidation, reported by Chen and White, giving primarily the branched product. **b)** The palladium catalyzed allylic oxidation giving primarily the linear product [18]. [L] = linear, [B] = branched.

Once the alkene is coordinated to palladium (*i.e.* a π-olefin-palladium complex) it becomes activated towards nucleophilic attack at the more substituted vinylic carbon. However using a novel bisulfoxide ligand **1** with palladium and benzoquinone (BQ) as oxidant, Chen and White reported successful Pd^II^-catalyzed allylic oxidation of terminal olefins (Scheme 5) [18]. Interestingly, the regioselectivity of the reaction could be controlled by using either **1** or DMSO in the reaction. 

**Scheme 6:**
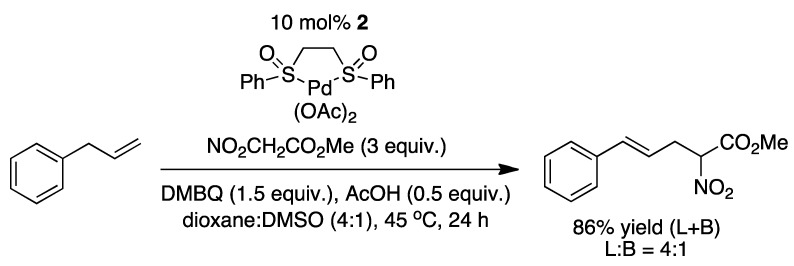
The conditions used by White with **2** to perform the intermolecular allylic C-H alkylation [14].

Young and White have used a similar sulfoxide ligand for intermolecular allylic C-H alkylation, using 2,6-dimethylbenzoquinone (DMBQ) as oxidant (Scheme 6) [14]. 

**Scheme 7:**
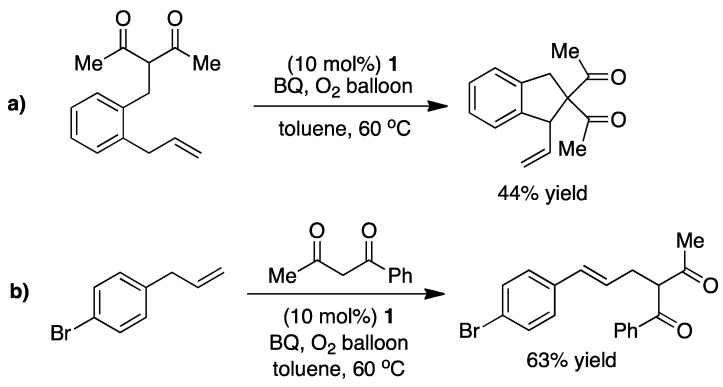
**a)** Intramolecular Pd-catalyzed allylic C-H alkylation. **b)** Intermolecular Pd-catalyzed allylic C-H alkylation. Both transformations were reported by Shi and coworkers [15].

Shi and coworkers has in a similar effort reported an intra/intermolecular allylic C-H alkylation facilitated by **1**. Interestingly the intermolecular version only produces the linear product (Scheme 7) [14,15]. 

These initial discoveries paves the way for further development of the palladium catalyzed allylic C-H alkylation. Recent results from related transformations, *i.e.* preparation of syn-1,3-amino alcohol motifs by allylic C-H amination [20], synthesis of complex allylic esters via C-H oxidation [21] or pyrrole synthesis via allylic sp^3^ C-H activation of enamines [22] underlines that this research area is constantly being expanded and that it receives considerable interest. However, to facilitate the development of more efficient catalysts and increase the scope of the reaction it would be advantageous to understand the reaction mechanism in detail. 

## 3. Elucidating the Mechanism

Recent research has shed some light on the mechanism and the catalytic cycle behind the allylic C-H alkylation although the reaction is still not completely understood. Young and White’s seminal studies showed that **2** effected rapid C-H cleavage of allylbenzene and provided good yields of a π-allylPd dimer [14]. The study showed further that the carbon nucleophiles with pKa < 6 (benzoylnitromethane, methyl nitroacetate and (phenylsulfonyl)nitromethane) gave alkylated products in good yields and useful regioselectivities. It was found that no reaction occurred in the absence of DMSO, which is known to be a π-acceptor ligand that activates π-allylPd complexes towards alkylation with malonates [23]. Furthermore, reactivity was severely reduced when adding stoichiometric Bu_4_NOAc, which may be a consequence of a shift in the position of the equilibrium between coordinately saturated and coordinately unsaturated Pd^II^ species [24]. A later study by Young and White [14] further underlines the importance of quinone/AcOH as a source of catalytic acetate base. These results supports a mechanism involving Pd^II^/sulfoxide-mediated C-H cleavage followed by DMSO/acetate promoted functionalization although the exact mechanistic details were not delineated. 

**Scheme 8:**
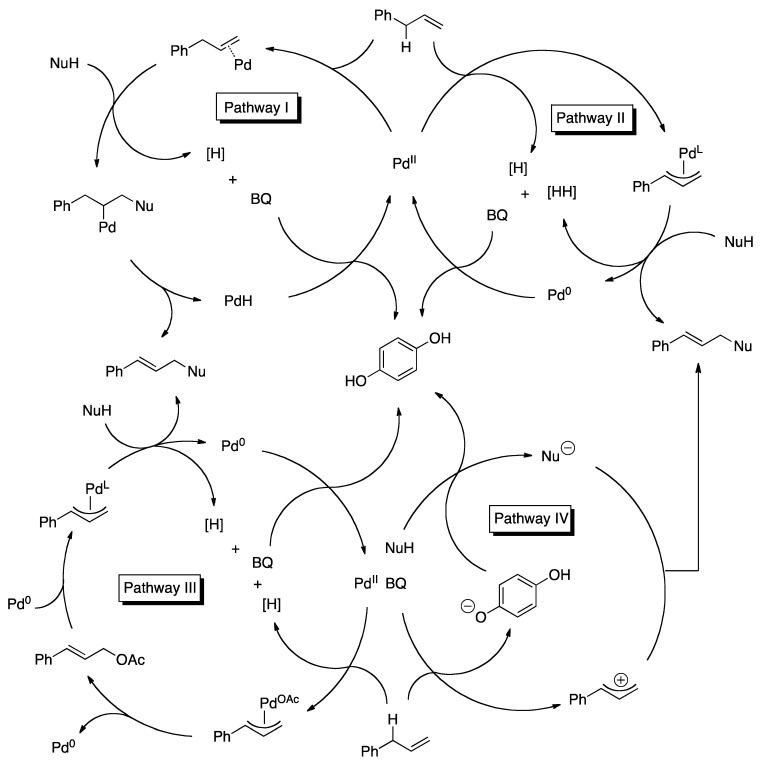
The proposed mechanisms by Shi and coworkers for allylic C-H alkylation [15].

**Scheme 9:**
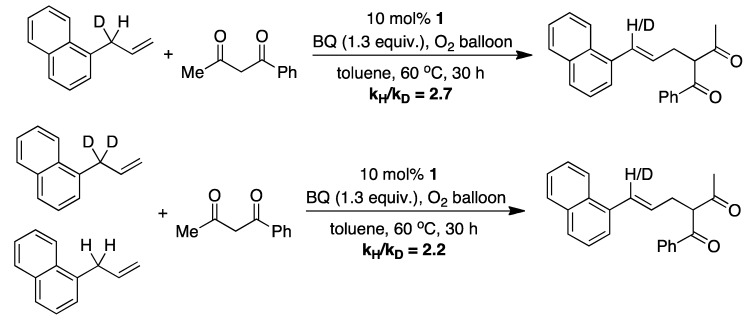
Isotope effect studies performed by Shi for the allylic C-H alkylation [15].

Shi and coworkers have proposed four different mechanistic pathways that govern the outcome of the allylic C-H alkylation (Scheme 8) [15]. Pathway I includes the Wacker-type process as the key step [25], however the mechanism does not correspond with the fact that only exo-type five/six-membered rings are formed in the intramolecular allylic C-H alkylation. Pathway II has a π-allylpalladium species as the key intermediate. This intermediate is formed by an electrophilic allylic C-H bond cleavage by a Pd^II^ catalyst and is the target for a nucleophilic attack by 1,3-dicarbonyl compounds or their enolate forms to afford the final product. Pd^0^ is then reoxidized by BQ to complete the catalytic cycle. Pathway III is postulated on the fact that small amounts of cinnamyl acetate was isolated in some cases and could serve as a key intermediate. This could be formed by C-H activation followed by acyloxylation, and then undergo Tsuji-Trost reaction to give the final product. However this mechanism proved to be wrong, since the traditional allylic alkylation with cinnamyl acetate under the described conditions did not form the desired product. Furthermore, a competition experiment involving a substrate with both an allylic acetate and an unsubstituted allyl groups a highly selective alkylation from the allylic C-H bond was observed, while the C-OAc bond was left intact. Pathway IV is based on work by Cheng and Bao, which reported a 2,3-dichloro-5,6-dicyanobenzoquinone(DDQ)-mediated oxidative coupling between diarylallylic C-H bond and active methylenic C-H bond [26]. Their results suggest a mechanism that involves oxidation of the olefin to an allyl cation by the quinone. However neither BQ or DDQ could perform the allylic alkylation in absence of Pd(OAc)_2_. Shi and coworkers concludes that pathway II is the most plausible mechanistic scenario. It is also pointed out, as with Young and White [14], that no base is necessary and that quinone acts as a proton acceptor as well as the oxidant. The proposed mechanism is further supported by determination of a significant kinetic isotopic effect (Scheme 9), which strongly suggests that the C-H/D bond is broken in the rate-determining step of the reaction. There was found to be no significant difference between intermolecular KIE and intramolecular KIE, thus suggesting that the C-H activation is *both* rate-determining (intermolecular competition between two substrates) *and* selectivity-determining (intramolecular between C-H and C-D). 

The formation of a stable π-allylpalladium complex in allylic C-H activation reactions is known from allylic C-H oxidation and amination [27,28]. Both the groups of White and Shi reports that only allylic substrates are tolerated, and for the intermolecular version only aromatic allylic substrates [14,15]. However, Shi and coworkers also attempted the intermolecular C-H alkylation with an aliphatic alkene but was only able to achieve a yield of 16%. 

## 4. Detailed Mechanistic Proposals

The mechanism behind the allylic C-H alkylation can be divided into three parts: C-H activation, nucleophilic attack and reoxidation. In this section we will discuss these three transformations individually while also including results obtained for closely related transformations.

### 4.1. C-H activation

**Scheme 10:**
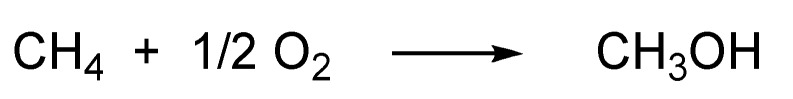
The partial oxidation of methane to methanol using molecular oxygen, which is a prototypical C-H activation reaction.

Activation of carbon-hydrogen bonds (C-H activation) is one of the simplest chemical transformations one can imagine. A “textbook” example is the conversion of methane to methanol (Scheme 10) that has the potential to allow utilization of the large reserves of natural gas present in remote locations from which transportation of the gas is not economically viable [29]. However, it is also one of the most challenging and there exists only very few efficient catalyst systems [12,30]. 

The fundamental challenge that needs to be addressed in these reactions are two-fold: i) The catalyst needs to have sufficient reactivity to engage the strong and unreactive C-H bond. ii) The catalyst should only carry out *one* C-H activation for each substrate molecule since the continued oxidation is usually not desirable. For methane itself the continued oxidation to the fully oxidized carbon dioxide (CO_2_) is energetically favored, thus the catalyst must be able to disengage after the first C-H activation. Interestingly, nature has been able to achieve this reaction by use of the enzyme methane monooxygenase, which features a complex di-iron active site with several oxygen-containing bridging ligands [31]. 

The most successful homogenous catalysts for methane oxidation to date is the classical Shilov system which involves a Pt^II^/Pt^IV^ redox couple in the catalytic cycle [13], and Pd-catalyzed formation of acetic acid from methane based on Pd^0^/Pd^II^ reported by Periana and coworkers [32]. 

**Scheme 11:**
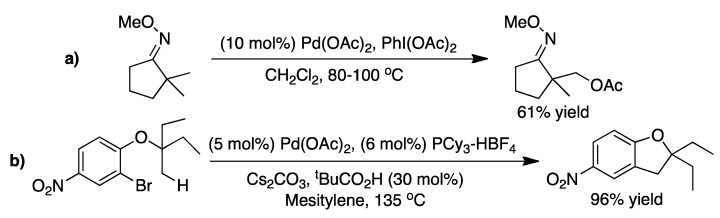
**a)** C-H activation in an alkane mediated by an *O*-methyl oxime group [35]. **b)** Ring closing procedure through sp^3^ C-H bond activation as reported by Fagnou and coworkers [36].

C-H activation is facilitated by the presence of a functional group in the substrate capable of coordinating to the catalyst. This have been reported by Sanford and coworkers with *O*-methyl oxime groups enabling sp^3^ C-H bond activation (Scheme 11a) [33,34]. Yu and coworkers have carried out dehydrogenation of alkyl groups by the use of palladium coordinating auxiliaries for carboxylic acids [35]. Fagnou and coworkers have used a slightly different approach is to have a functionality, which is ideal for oxidative addition built into the reagent in close proximity to the C-H bond. The use of halide substituents in aromatic systems leads to the formation of Pd^II^ complexes by oxidative addition in which the palladium is in close proximity to an sp^3^ C-H bond. The C-H bond can then be activated by proton abstraction performed by a carbonate or pivalate ligand on palladium, giving it character of an electrophilic substitution mechanism (Scheme 3), and ultimately ring closing is performed (Scheme 11b) [36,37,38]. A similar example for the synthesis of benzocyclobutenes have been reported by Baudoin and coworkers [39]. 

**Scheme 12:**
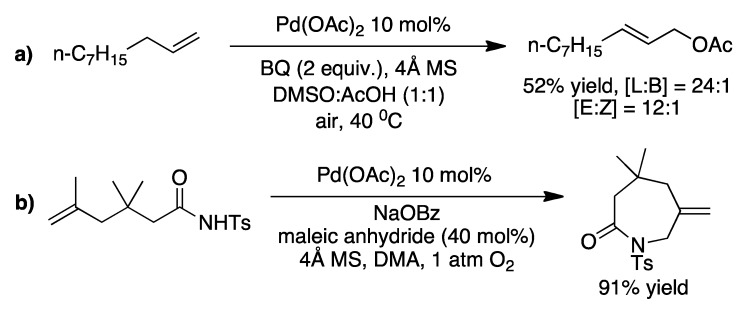
**a)** An example of the intermolecular oxidation reported by Chen and White [18]. **b)** The intramolecular amination reported by Liu and coworkers, which leads to seven-membered cyclic amides [42].

Activating sp^2^ C-H bonds, alkenes, is less difficult and there have been reported intermolecular oxidation [18], as well as intermolecular and intramolecular aminations (Scheme 12) [24,40,41,42].

**Scheme 13:**
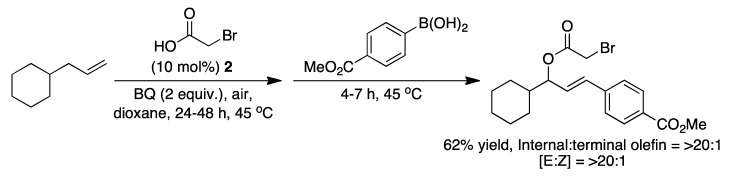
The sequential C-H bond transformation of an alkene to an E-arylated allylic ester [44].

Heteroatoms in the substrate that coordinates to palladium can also promote the C-H activation. An example of this is the use of another olefin in the substrate that coordinates to palladium, so a η^2^-η^2^-complex is formed before the C-H cleavage generates a η^3^-η^2^-complex [43]. Delcamp and White have reported sequential C-H bond transformations with alkenes, where an allylic C-H oxidation is followed by a vinylic C-H arylation [44]. In a one-pot Pd^II^/sulfoxide-catalyzed reaction α-olefins can be converted to E-arylated allylic esters with high regio- and stereoselectivities (Scheme 13). 

Because of the acidic nature of a proton in the terminal position of an alkyne, it can be difficult to determine whether an actual C-H activation is taking place or not in a given palladium-catalyzed reaction with an alkyne. Therefore C-H activation of alkynes will not be mentioned here. The coupling of alkynes with various reagents by the Sonogashira reaction with palladium is well known and has been reviewed thoroughly elsewhere [45]. 

C-H bonds in aromatic systems are relatively easy to activate and the research field has received considerable attention, which has lead to interesting applications of the generally used palladium-diacetate catalyst. As the bis-*ortho*-arylation of biphenyl diamines published by Stahl and coworkers [46] or the cyclization of aryl complexes by intramolecular C-H activation [47]. The use of nitrogen atoms for coordination to Pd in the arene reagents has been well documented by Sanford and coworkers [48,49,50,51], especially the regioselective C-H activation in benzo[*h*]quinolone systems [52,53,54]. Some successful C-H activations with indoles have also been reported [55,56,57], as well as with azoles [58]. 

**Scheme 14:**
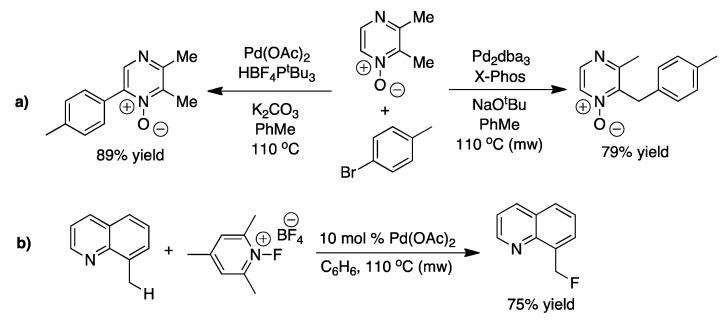
**a)** The selective phenyl or benzyl C-H activation with either the Pd(OAc)_2_ or Pd_2_dba_3_ catalyst [59]. **b)** Shows the benzylic fluorination reported by Sanford and coworkers [60].

Catalysts that are capable of activating a C-H bond in the benzylic position is often also capable of performing C-H activation of alkenes. As a result benzylic C-H activation reactions have been reported together with alkene C-H bond activation in several cases [40,61,62,63]. However, some benzylic C-H activation have been reported without an olefin being present in the reagent, for example the selective sp^2^ or benzylic sp^3^ arylation with either acetate or dibenzylideneacetone ligands on the palladium catalyst (Scheme 14a) [59]. Also fluorination of the benzylic position on methylquinolines has been successful with palladium-diacetate (Scheme 14b) [60]. 

**Scheme 15:**
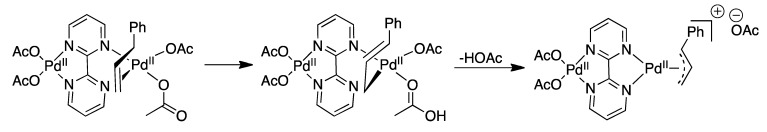
A hydrogen abstraction could be facilitated by an acetate ligand on the palladium catalyst [63].

Abstraction of a benzylic hydrogen atom also takes place in the allylic C-H alkylations reported by White [14] and Shi [15], because the reaction only works with aromatic allylic substrates. The exact details of how this C-H abstraction takes place to form the allyl intermediate is not known at the present time. Mechanisms for similar C-H activations have been suggested, for example in the allylic acetoxylation of olefins by a palladium-bipyrimidine complex reported by Bercaw and coworkers (Scheme 15) [63]. The mechanism suggested by Bercaw and coworkers is based on NMR spectroscopy and results from deuterium-labelling experiments. 

This mechanism in which an acetate ligand on the palladium enables hydrogen to be abstracted as a proton is also the C-H activation mechanism in a proposed mechanism for intermolecular allylic C-H amination [28] and a published catalytic cycle for allylic C-H oxidation [64]. An acetate ligand also acts as the proton abstractor in the mechanism for a direct intramolecular allylic amination reported recently [65]. The participation of an acetate ligand as proton abstractor in these listed mechanisms categorizes them as electrophilic substitution mechanisms for C-H activation (Scheme 3) [12]. Furthermore the powerful synergy between transition metals and carboxylate or carbonate ligands in facilitating C-H bond activation has been explored by computational chemistry [66]. It is pointed out that the catalyst complex works as a Lewis acidic metal centre and an intramolecular base simultaneously. Results by Liu and coworkers with allylic C-H amination supports a different mechanism, where the nucleophile, an imidate, is coordinated to palladium when the C-H activation occurs [67]. 

### 4.2. Nucleophilic attack

In the Tsuji-Trost reaction a wide array of nucleophiles can be utilized in the reaction, but soft stabilized carbon nucleophiles are commonly used in the reaction, like β-dicarbonyls, cyanoesters and malononitriles [68]. Other derivatives containing electron-withdrawing groups, such as nitro, sulfonyl and iminyl groups can also be used. Heteroatom nucleophiles are also applicable in the allylic substitution, such as amines, imides, amides and aryloxides. Various cases of the use of alkali metal enolates used in the reaction are also reported. Furthermore reactions with boron-, silicon-, tin- and zinc-enalotes have been reported [69,70,71]. 

The allylic C-H alkylation has been reported performed with soft nucleophiles, being various β-dicarbonyl compounds as reported by Shi and coworkers [15] or with benzoylnitromethane, methyl nitroacetate and (phenylsulfonyl)nitromethane compounds as reported by Young and White [14]. As mentioned earlier it was found, by Young and White that the nucleophile should have a pK_a_ below 6 to work efficiently. It has been postulated that this is linked to the use of BQ as the oxidant agent, since the oxidation potential of BQ is pH dependent [72]. There is a protonation involved in the redox process to generate hydroquinone and as a result in catalytic applications acidic conditions have to be used, which limits the scope of useable nucleophiles in the reaction. Another explanation for the pK_a_ limitation is, as reported by Liu and coworkers, the need for the nucleophile to coordinate to the palladium complex to facilitate the C-H bond cleavage [73]. 

It is also a possibility that BQ is acting as a ligand in the π-allylpalladium complex and facilitates functionalization, as found by White and coworkers for allylic C-H oxidation [64]. If a catalytic system relying on direct aerobic oxidation of palladium is used no BQ is needed for reoxidation and maleic anhydride can be utilized to promote the nucleophilic attack instead of BQ [73]. The same replacement of BQ has been performed with a nitrogen based bidentate ligand for aerobic catalysis of allylic C-H acetoxylation [74]. It has also been shown that the nucleophilic attack can be facilitated by addition of a Lewis acid in the form of a Cr^III^-salen complex [75]. 

Further research should focus on developing reaction conditions that would allow nucleophiles with a pK_a_ above 6 thereby expanding the substrate scope. Whether the nucleophilic attack in the allylic C-H alkylation takes place by an inner-sphere or outer-sphere mechanism has not been determined conclusively. The formation of the stable π-allylPd complex supports an outer-sphere mechanism, but this could simply be a resting state for the catalyst. 

### 4.3. Reoxidation

**Scheme 16:**
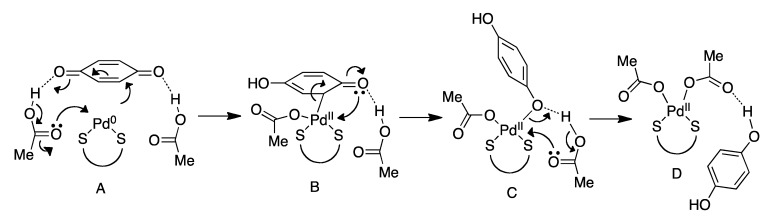
The oxidation of Pd^0^ to Pd^II^ by BQ as reported by Poli and coworkers [65].

The oxidation of Pd^0^ to Pd^II^ by use of BQ or similar oxidants has been utilized in the majority of reactions relying on allylic C-H activation. This oxidation was investigated recently in a computational study by Poli and coworkers (Scheme 16) [65]. The free energies of the structures **A**-**D** and their intermediates were determined by DFT calculations. The starting structure **A** undergoes a conjugate addition of Pd^0^ to BQ to give intermediate **B** through an energy barrier of +17.9 kcal/mol. This oxidizes Pd^0^ to Pd^II^ and the palladium is coordinated to the ligand, AcO^-^ and BQ. The last interaction is through the C atom α to the carbonyl and is a rather long Pd-C bond of 2.19 Å. The hydrogen bonding between the second AcOH and BQ is crucial for the next step **B**-**C**, as it lowers the energy barrier significantly (by 14 kcal/mol). The phenol structure is obtained in **C**, where the BQ carbonyl acts as the 2e^-^ donor. The final step **C**-**D** gives the second BQ protonation and the regenerated Pd^II^ catalyst with two AcO^-^ ligands. These results by Poli and coworkers show that the two AcOH molecules release protons, act as ligands and furthermore influences the redox potential of the palladium. 

**Scheme 17:**
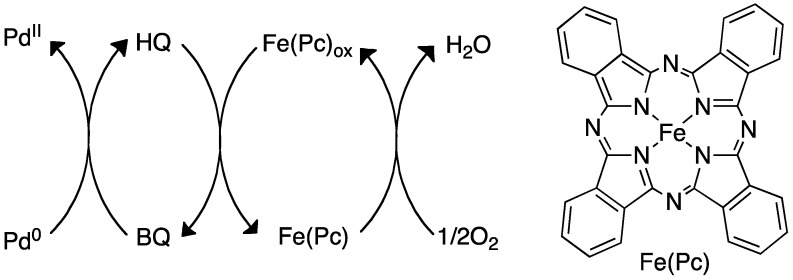
Reoxidation of Pd^0^ by use of BQ, iron and O_2_ [80].

Poli and coworkers reported this oxidation of Pd^0^ to Pd^II^ by BQ in an allylic amination, but a similar mechanism could be operating in the alkylation. The oxidation of the catalyst can be imagined performed in various ways [76] and several methods have been reported. The oxidation can be achieved by use of Cu(OAc)_2_ and oxygen, as reported by Loh and coworkers for couplings of alkenes with acrylates [77] or with a combination of catalytic amounts of BQ and MnO_2_ [78]. Recent results by Liu and coworkers shows that allylic C-H amination can be achieved by use of the strong soluble oxidant PhI(OPiv)_2_ [67]. The reoxidation of Pd^0^ can also be accomplished with an NPMoV/O_2_ system [79] or even via a triple catalytic system relying on iron (Scheme 17) [80]. 

It is always desirable to reoxidize the catalyst by using dioxygen or even air. The reoxidation with dioxygen has been accomplished in a few cases of C-H activation, such as allylic C-H acetoxylation [74], indole synthesis [81] and iminoannulation of internal alkynes [82]. The reoxidation with air has been reported for direct dehydrogenative annulation of indole-carboxamides with alkynes [83]. 

## 5. Conclusions and Outlook

Research into the mechanism behind the palladium catalyzed allylic C-H alkylation is limited at the present time, but there is an agreement about the key intermediate being the π-allylpalladium species [14,15]. More detailed studies into the mechanism could expand the substrate scope of this allylic C-H alkylation and facilitate the development of more specific and efficient catalysts. Furthermore the establishment of a general enantioselective protocol would be highly advantageous and recent results, *i.e.* the use of a chiral sulfoxide ligand [84] or a chiral Lewis acid [75], illustrates that this goal can be reached in the near future. A better understanding of the fundamental mechanistic aspects of C-H activation could also lead to implementation of C-H activation procedures in other palladium-catalyzed reactions, as shown recently with the combination of a C-H activation and a Suzuki-coupling [85]. 
